# Electrical activity at the AlN/Si Interface: identifying the main origin of propagation losses in GaN-on-Si devices at microwave frequencies

**DOI:** 10.1038/s41598-020-71064-0

**Published:** 2020-08-25

**Authors:** Micka Bah, Damien Valente, Marie Lesecq, Nicolas Defrance, Maxime Garcia Barros, Jean-Claude De Jaeger, Eric Frayssinet, Rémi Comyn, Thi Huong Ngo, Daniel Alquier, Yvon Cordier

**Affiliations:** 1 GREMAN UMR-CNRS 7347, Université de Tours, INSA Centre Val de Loire, 16 rue Pierre et Marie Curie, BP 7155, 37071 Tours Cedex 2, France; 2 CNRS-IEMN – Université de Lille, UMR8520, Av. Poincaré, 59650 Villeneuve d’Ascq, France; 3 Université Côte d’Azur, CNRS, CRHEA, rue B. Gregory, 06560 Valbonne, France

**Keywords:** Applied physics, Electronics, photonics and device physics, Materials for devices, Materials chemistry, Chemistry

## Abstract

AlN nucleation layers are the basement of GaN-on-Si structures grown for light-emitting diodes, high frequency telecommunication and power switching systems. In this context, our work aims to understand the origin of propagation losses in GaN-on-Si High Electron Mobility Transistors at microwaves frequencies, which are critical for efficient devices and circuits. AlN/Si structures are grown by Metalorganic Vapor Phase Epitaxy. Acceptor dopant in-diffusion (Al and Ga) into the Si substrate is studied by Secondary Ion Mass Spectroscopy and is mainly located in the first 200 nm beneath the interface. In this region, an acceptor concentration of a few 10^18^ cm^-3^ is estimated from Capacitance–Voltage (C–V) measurements while the volume hole concentration of several 10^17^ cm^-3^ is deduced from sheet resistance. Furthermore, the combination of scanning capacitance microscopy and scanning spreading resistance microscopy enables the 2D profiling of both the *p*-type conductive channel and the space charge region beneath the AlN/Si interface. We demonstrate that samples grown at lower temperature exhibit a *p*-doped conductive channel over a shallower depth which explains lower propagation losses in comparison with those synthesized at higher temperature. Our work highlights that this *p*-type channel can increase the propagation losses in the high-frequency devices but also that a memory effect associated with the previous sample growths with GaN can noticeably affect the physical properties in absence of proper reactor preparation. Hence, monitoring the acceptor dopant in-diffusion beneath the AlN/Si interface is crucial for achieving efficient GaN-on-Si microwave power devices.

## Introduction

GaN-based High Electron Mobility Transistors (HEMTs) are of great interest for the next generation of high frequency telecommunication and power switching applications owing to their large band gap, high breakdown field, high electron mobility, good thermal conductivity, high output power density, etc. Furthermore, GaN-on-Si HEMTs offer the possibility to fabricate low cost devices since high quality Si wafers with a large diameter are commercially available at low price. However, the direct epitaxial growth of GaN-on-Si is difficult due to high lattice mismatch and large thermo-elastic strain and can simultaneously induce GaN cracking and formation of undesirable Si_3_N_4_ phase at the GaN/Si interface or Si substrate etching by Gallium. Such difficulties have been overcome by intercalating suitable AlN nucleation layers between Si and GaN/Al(Ga)N based buffer layers. Besides, understanding and monitoring the electrical activity at the AlN/Si interfacial junction is crucial for improving high frequency devices and power switching transistor performances. Indeed, it is still a major concern to achieve high electrical resistivity in epilayers, especially for low RF losses in high frequency operation transistors and circuits for applications like 5G^1^. Not only the use of high-resistivity substrates is necessary^2^, but also the achievement of both sufficient crystal quality and electrical resistivity of AlN/Si interface is required. Different phenomena have been reported as possible origins of parasitic conductivity and propagation losses: the diffusion of dopant species into the Si substrate^3–5^, the formation of an inversion layer at the AlN/Si interface^6–9^ as well as degraded crystal quality^7,10^. In this context, the combination of several techniques is necessary to know the composition of the interface and its electrical behavior. In the present study, the interest of cross-sectional electrical analysis using an atomic force microscope (AFM) such as scanning capacitance microscopy (SCM) and scanning spreading resistance microscopy (SSRM) becomes clear. The observed active dopant distribution in Si after growth of AlN correlated with the chemical analysis of Secondary Ion Mass Spectroscopy (SIMS) gives a spatially resolved electrical profile not accessible with conventional C-V and Hall effect measurement techniques. All these results correlate well with the sheet resistance and RF propagation losses.

## Results

### Sample description

**Figure 1:**
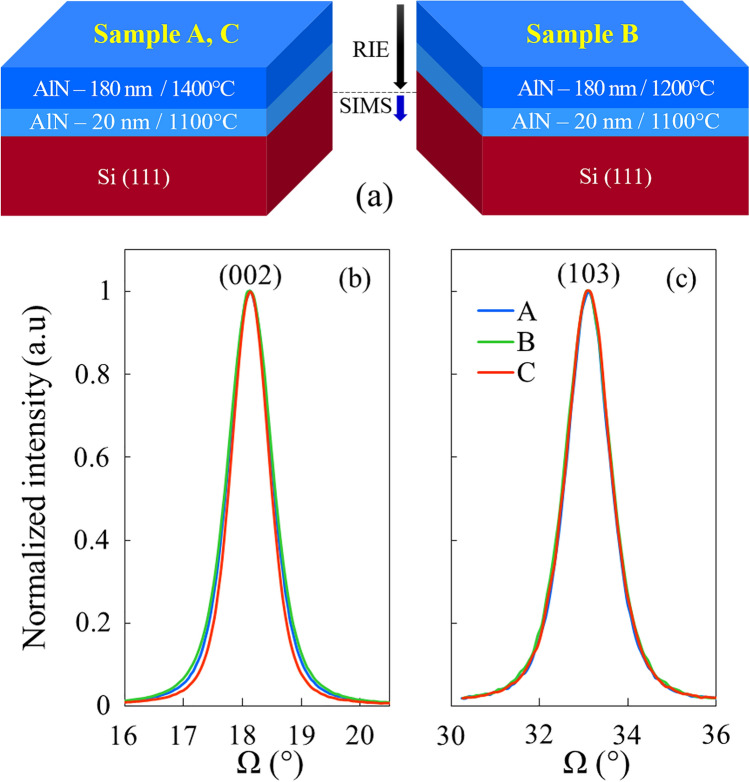
(**a**) Structure of samples A, B and C. The nominal growth rate for the AlN buffer was 4.3 nm/min for the first 20 nm and 8.7 nm/min for the remaining 180 nm. XRD scans of the (**b**) symmetric (002) and (**c**) asymmetric (103) peaks for samples A, B and C.

**Figure 2:**
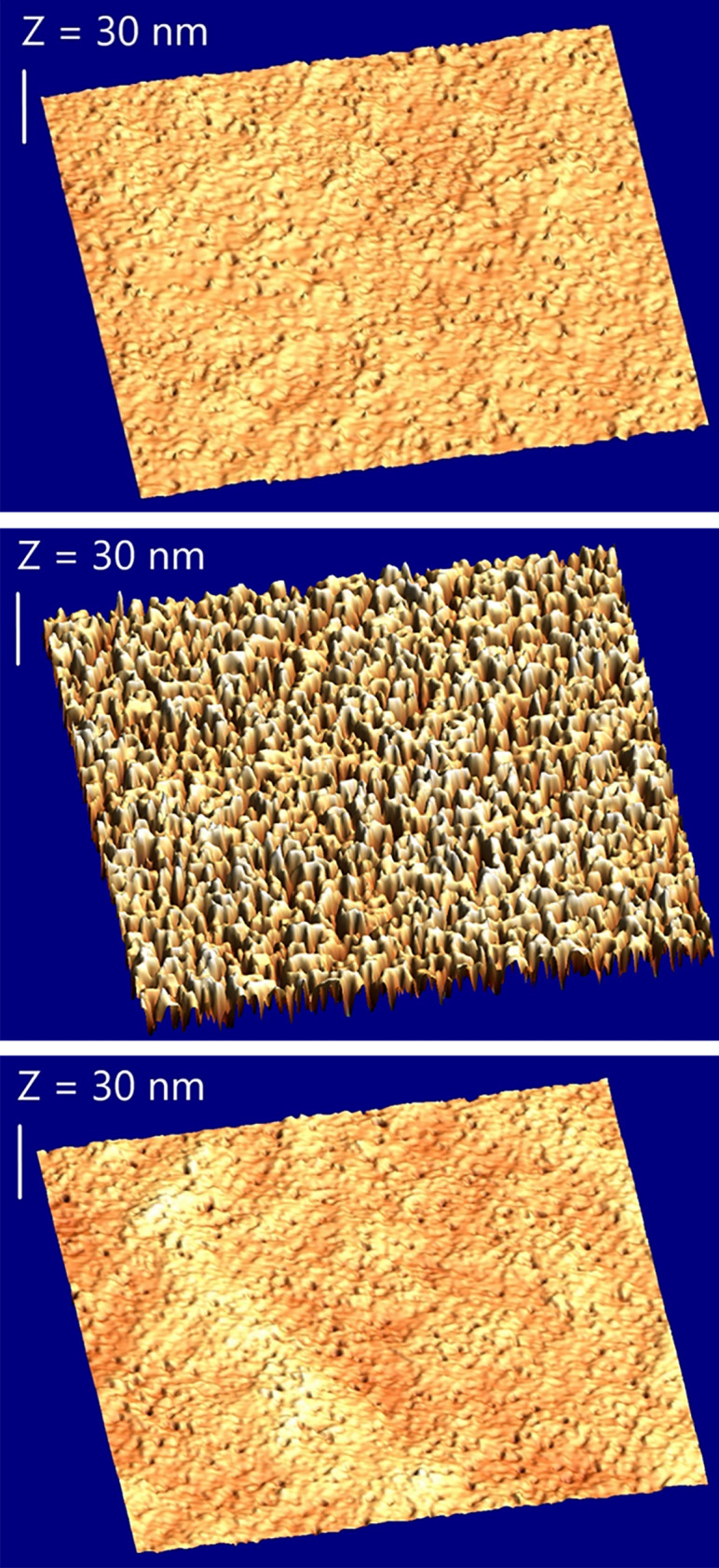
From top to bottom, AFM 3D views of the surface of samples A, B and C (5 µm × 5 µm area).

The samples are grown by Metalorganic Vapor Phase Epitaxy (MOVPE) in a Close Coupled Showerhead (CCS) system^11^. The Si (111) substrates are 2 in. diameter 500 µm thick intrinsic with an initial resistivity higher to 5000 Ω.cm (*n*-type). The epilayers consist in non-intentionally doped AlN buffers (Fig. 1a) which are the base of epitaxial stacks for GaN based structures on Si substrate^12^. After Si oxide removal under H_2_ around 1000 °C, a thin 20 nm nucleation layer is grown at low temperature (1100 °C thermocouple setpoint, real temperature around 900 °C). The nucleation is performed using a NH_3_ preflow to limit the risk of Al diffusion towards the substrate^13^. Then, the substrate temperature is rapidly increased to grow the AlN film with a total thickness up to 200 nm. The main features of the studied samples are summarized in Table 1. Samples A and C were grown using the same setpoints (real temperature of 1150 °C) whereas sample B was grown at lower temperature (real temperature of 1000 °C). Figure 1b, c show the symmetric (002) and asymmetric (103) X-ray diffraction (XRD) omega scans respectively performed to evaluate the crystal quality. Figure 2 shows the surface morphology of these samples (scan area is set to 5 × 5 µm^2^). As reported in Table 1, the growth at higher temperature results in smoother surface with reduced root mean square (RMS) roughness as well as slightly enhanced crystal quality with smaller full width at half maximum (FWHM) of symmetric (002) and asymmetric (103) XRD peaks. However, as shown in Fig. 2, this is not sufficient to eliminate pinholes usually attributed to dislocations.

**Table 1:** Growth parameters and physical characteristics of the AlN buffer on Si substrate for samples A, B and C.

	Growth temperature setpoint (°C)	Total thickness (nm)	AFM rms (nm)	XRD FWHM (°)	
	–	–	–	(002)	(103)
Sample A	1400	210	0.8	0.76–0.85	1.16–1.36
Sample B	1200	229	2.4–3.2	0.89–0.98	1.2–1.4
Sample C	1400	198	0.6–0.7	0.77–0.81	1.18–1.24

### SIMS measurements

**Figure 3:**
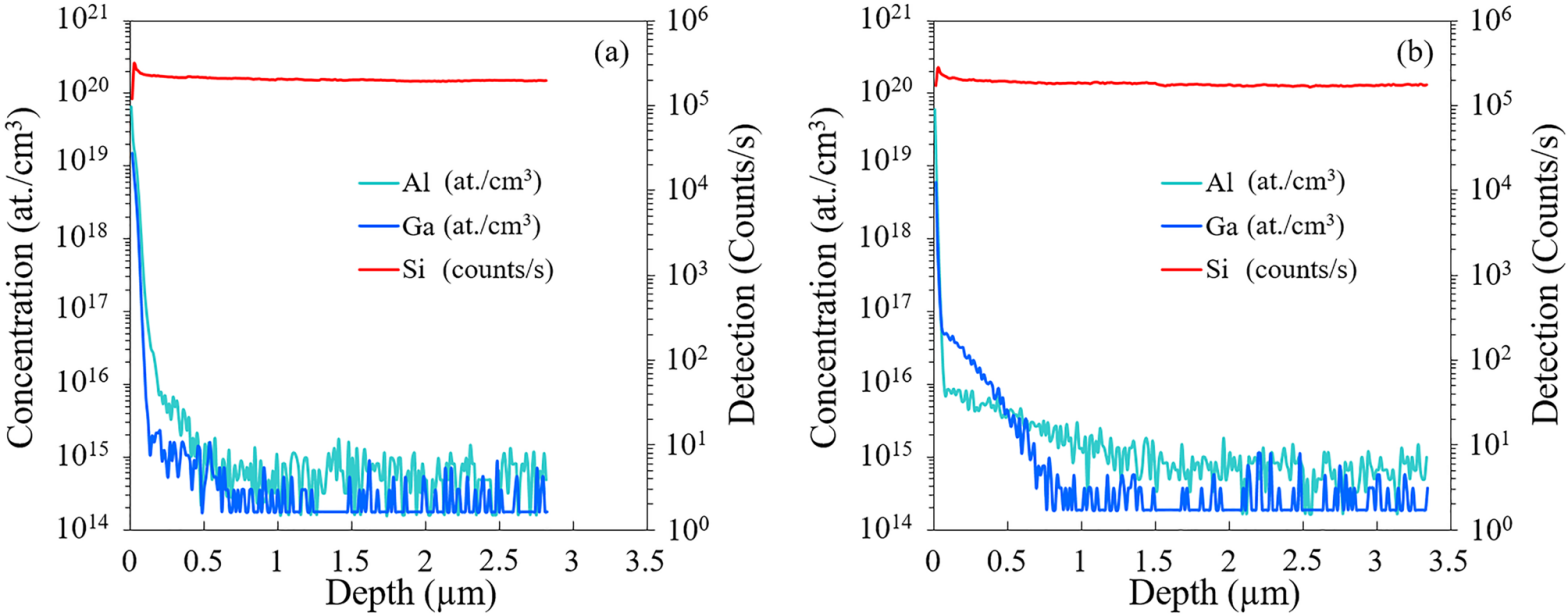
SIMS profiles of Al and Ga concentrations as a function of depth from 75 ± 15 nm and 73 ± 28 nm beneath the AlN/Si interface toward interior volume of *n*-Si substrate for sample B and A, respectively.

Since the prospect of this work is to design efficient GaN-on-Si microwave power devices, it is crucial to carefully control all the process steps. This includes among other things the preparation of the MOVPE reactor to eliminate Ga and Al traces which can diffuse into the substrate in a new process step and the control of process temperatures/duration for the AlN nucleation and buffer growth in order to master the crystal quality and the electrical activity at nanoscale at the material junction, etc. The typical preparation of the MOVPE reactor consists in scrapping the showerhead to remove excess of material deposited on it and injecting hydrogen and ammonia while heating the susceptor. SIMS is an appropriate technique to explore these interrelated effects in the sense that one can access to the spatial distribution of Ga and Al species in Si. However, the partial species ionization (acceptor energies around 70 meV) and the possibility to occupy other sites than acceptors’ make the direct estimation of electrically active acceptor concentrations impossible. As schematized in Fig. 1a, prior to SIMS measurements, the whole AlN layer was removed using reactive ion etching (RIE) in order to suppress the influence of Al from this latter. After the RIE process, removed material thickness (including AlN layer and Si) is estimated with a stylus profilometer to 283 ± 28 nm and 305 ± 15 nm for samples A and B, respectively. Figure 3 shows the profiles of Al and Ga concentrations into *n*-Si substrate for sample A and B. Ga and Al have thermally diffused beneath the AlN/Si interface as can be expected because these two acceptor dopants have high diffusivities^14–18^. Ga originates from minuscule traces after previous growths in spite of the reactor preparation consisting in scrapping the showerhead and heating the susceptor under ammonia and hydrogen flows. However, the estimation of the real [Ga] and [Al] concentrations at the AlN/Si interface is a bit tricky for several reasons. First, about 70 nm of Si have been etched away prior to measurements, yet the profiles evolve very rapidly in this region. Second, it may be that the profile is influenced a priori by the push-in effect associated with an incomplete removal of Al during AlN RIE etching. The noisy behavior with a difficulty to reach the detection limit at depths superior to 1 µm indicates that this memory effect can exist, at least for low residual [Al] amounts. However, this effect cannot account for [Ga]. Furthermore, Fig. 3a shows that, within the first 100 nm probed by SIMS in sample B, the [Ga] level initially at about 10^19^/cm^3^ fell rapidly bellow the [Al] level contrary to sample A (Fig. 3b) and sample C (not shown). This confirms that despite a lack of accuracy, differences depending on the growth process really exist in terms of [Ga] and [Al] concentrations in the vicinity of the AlN/Si interface. As already mentioned, these dopant profiles drop down rapidly before they slowly continue to decrease down to depths of several hundreds or thousands of nm. For instance, the Aluminum concentrations estimated from SIMS plots at about 100 nm depth (170 nm from AlN/Si interface) are [Al] = 8 × 10^15^/cm^3^ and [Al] = 1 × 10^16^/cm^3^ for sample A and B, respectively. Furthermore, separate measurements made on sample C showed [Al] ~ 1 × 10^16^/cm^3^, which means that at least at about 170 nm underneath the interface Al concentration is similar in spite of changing the AlN buffer growth temperature from 1100 °C for sample B to 1150 °C for samples A and C. However, it seems that even if the signal is noisy, it saturates at a few 10^14^/cm^3^ at about 1500 nm, 600 nm and 1600 nm for samples A, B and C, respectively. On the contrary, at a depth of about 100 nm Gallium concentrations increase by more than one order of magnitude and become predominant for sample A ([Ga] = 6 × 10^16^/cm^3^) compared with sample B ([Ga] = 2 × 10^15^/cm^3^). Having in mind growth temperatures, profiles are pretty shallow indicating that the dopant source is limited, which indicates that the source of impurity may be chamber memory. The presence of Gallium beneath the AlN/Si interface may be due to a residual amount of pure Ga, or Ga sources like GaN or Tri-Methyl-Gallium (TMGa) metalorganic precursor in the CCS reactor^19^ diffusing into the Si substrate during the substrate oxide removal and AlN nucleation steps. Ga is probably introduced early in the process before the coalescence of the AlN buffer layer crystallographic grains occurring after few nm. Indeed, it is worth mentioning that during its thickening the AlN buffer can progressively act as a diffusion barrier slowing down Ga thermal diffusion into the Si substrate. On the other hand, Si thermal diffusion into the insulating AlN buffer may only induce a negligible effect on electrical properties because of the very large ionization energy of donors^20^. However, the float zone grown substrate is intrinsic so that chemical identification of the residual *n*-type doping is difficult. Nevertheless, it is clear from Fig. 3 that the Ga and Al species have diffused to different depths in the Si.[Al] concentration starts to saturate at a few 10^14^/cm^3^ for a depth around 1.6 µm underneath the AlN/Si interface in Sample A whereas it is about 700 nm in sample B. Furthermore, [Ga] concentration reaches the detection limit around 2 × 10^14^/cm^3^ for depths of 850 nm and 700 nm in samples A and B respectively. Altogether, the SIMS profiles indicate that the metallurgical junction is probably located less deeply in sample B than in samples A and C.

### Electrical measurements

**Figure 4:**
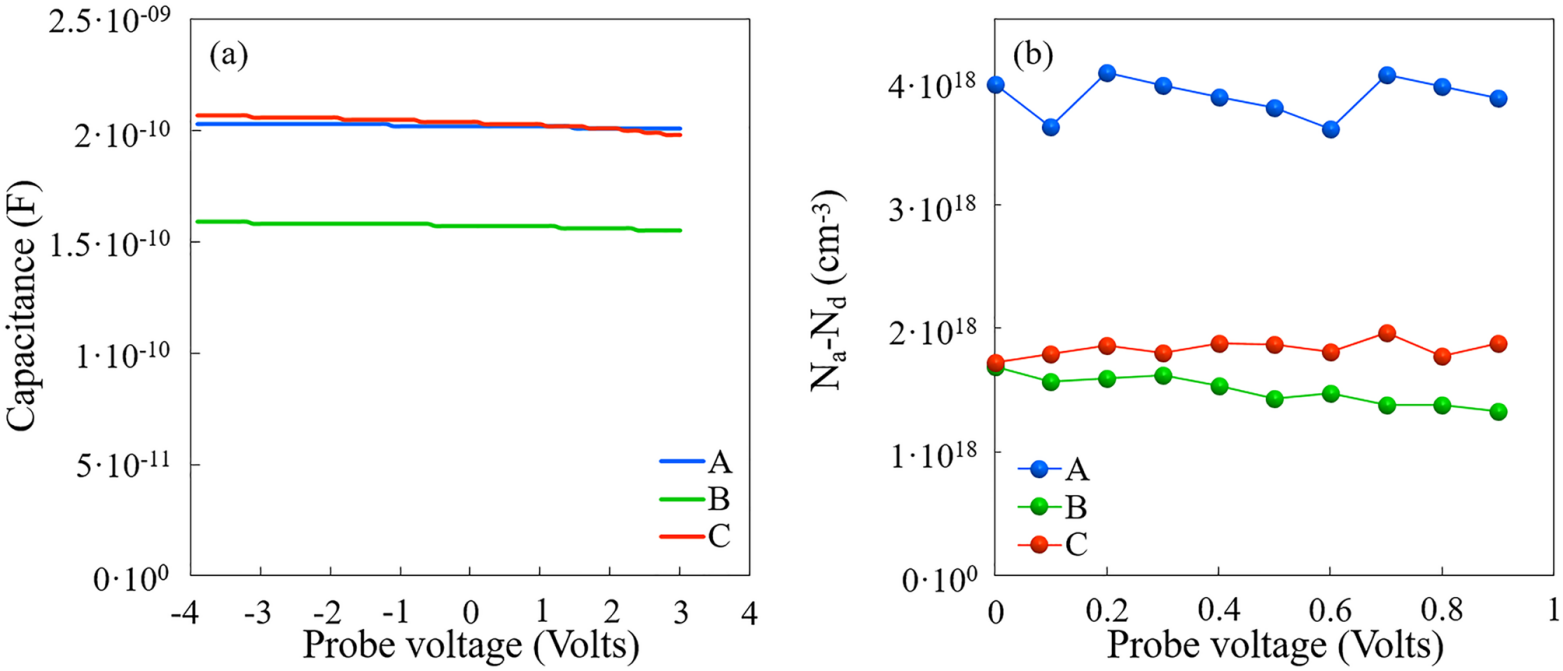
(**a**) Capacitance voltage measurements at 10 kHz by mercury probe contacts for sample A, B and C. (**b**) Net acceptor concentration in Si.

The SIMS measurements have shown the presence of Ga and Al having diffused into the Si substrate. However, at this stage, the electrical activity of acceptors is not known. For this reason, C-V measurements have been performed at 10 kHz with a Mercury probe at the surface of AlN. Figure 4a shows the profiles obtained when a bias is applied to the central dot. For biases up to 3 V, similar values of capacitance are obtained for samples A and C, which have similar thicknesses smaller than that of sample B. Taking into account a relative permittivity of AlN around 9, it appears that the capacitance corresponds to the thickness of the AlN layer in samples A and C since a planar capacitor is formed between Mercury at the surface of the highly resistive AlN and the doped regions in Si. However, the larger thickness of sample B accounts only for a part of the difference in capacitance. Possible reasons are a lower permittivity of AlN related to the worse crystal quality, a depletion of carriers underneath the AlN layer, or the effect of a noticeably larger series resistance due to a larger resistivity. As shown in Fig. 4a, the positive change of bias slightly reduces the capacitance, which is compatible with the presence of a region populated with a large amount of holes close to the AlN/Si interface. In case of *p*-type doped Si, a positive bias repels holes and widens the depletion region thus reducing the capacitance. As shown in Fig. 4b, the net acceptor concentration N_a_-N_d_ is found by using the well-known Eq. ^21^:

1 $${\text{N}}_{{\text{a}}} - {\text{N}}_{{\text{d}}} \,({\text{V}}) = 2/\left( {{\text{q}} \cdot \varepsilon \cdot \left( {{\text{d}}\left( {1/{\text{C}^{2}}} \right)/{\text{dV}}} \right)} \right),$$

where q is the electron charge and ε the Si permittivity. This leads to acceptor concentrations of 4 × 10^18^, 1.6 × 10^18^ and 1.9 × 10^18^ cm^-3^ for samples A, B and C, respectively, near the AlN/Si interface. Like the others electrical characterizations described below, the measurements of sample C evidence a lower doping compared to sample A in spite of identical growth conditions. This will be discussed in the last section of this paper.

From the measurements we deduced that a lot of acceptors are present underneath the AlN layer, with volume concentrations and maybe distances from the interface depending on the growth process. However, the difficulties to deplete the interface region from holes makes the estimation of the total number of acceptors impossible with the present setup. To obtain a rough estimation of the number of hole carriers, the sheet resistance was measured using a contactless Eddy current system at 980, 9560 and 3190 Ω/sq for samples A, B and C respectively. The hole mobility deduced from Hall effect measurements performed on samples consisting of the 20 nm low temperature grown AlN nucleation layer of the studied samples is between 250 and 300 cm^2^/V·s. Assuming that only holes contribute to the conductivity, such mobilities would lead to hole sheet carrier densities of 2.1–2.5 × 10^13^, 2.1–2.6 × 10^12^, and 6.5–7.8 × 10^12^ cm^-2^ for samples A, B and C, respectively. However, none of the present characterizations give a profile of the actual net acceptor or carrier concentrations. To do so, SCM and SSRM measurements have been undertaken.

### SCM and SSRM measurements

**Figure 5:**
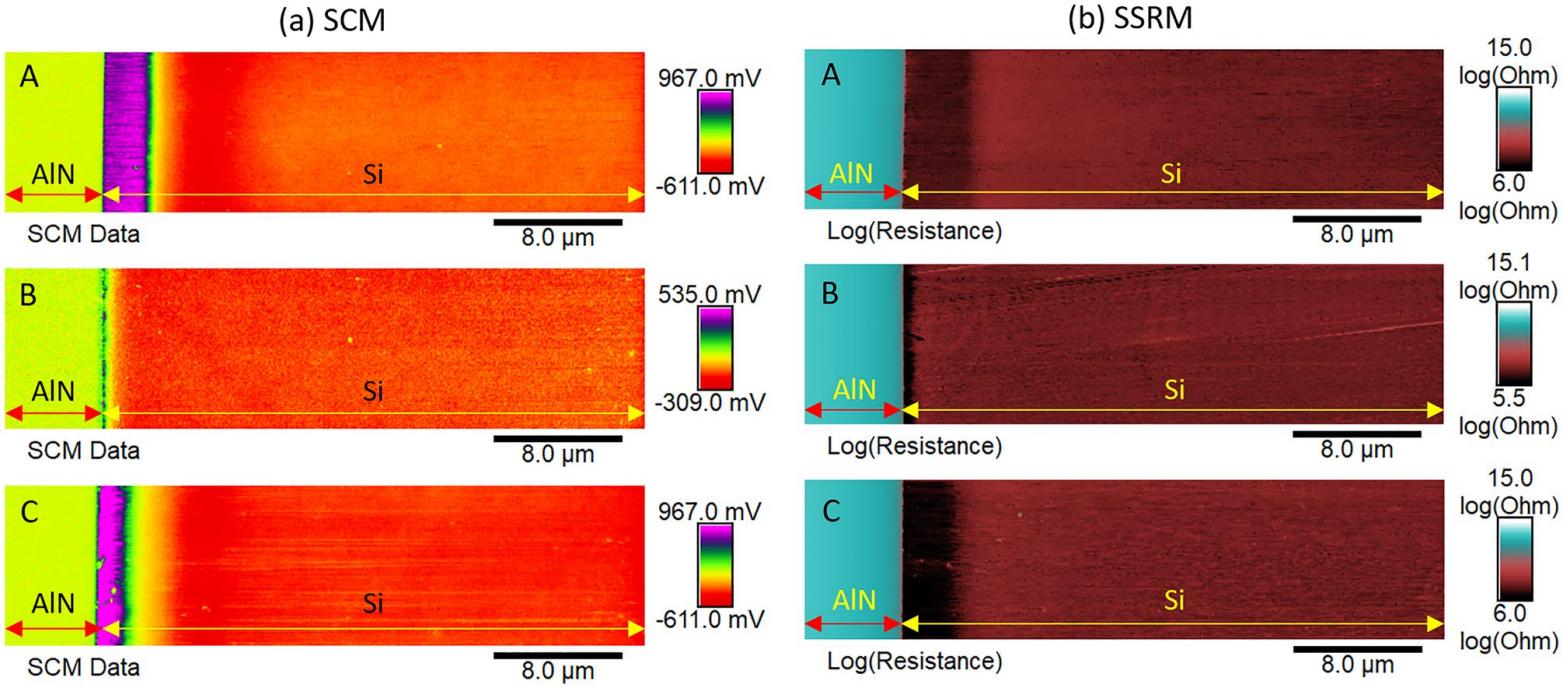
Electrical activity at the AlN/Si interface for samples A, B and C: (**a**) SCM data are proportional to the product of dC/dV-phase and dC/dV-amplitude and (**b**) SSRM data correspond to the measured resistance (Ohm) on logarithmic scale.

SCM and SSRM techniques are deployed to examine the electrical activity at the AlN/Si interface. Topography images are acquired at the same time as capacitance and resistance maps enabling direct correlation between local topography and electrical properties and with the spatial resolution. Topography, deflection error, dC/dV-phase and dC/dV-amplitude maps are shown in supplementary figures S3 and S4. The estimated root mean square roughness of Si beveled region RMS_Si_ is equal to 3.1, 2.5 and 3.7 nm while that of the AlN beveled region RMS_AlN_ is about 2.7, 2.4 and 2.6 nm for samples A, B and C, respectively. These low values of RMS roughness after the mechanical polishing step are favorable for electrical measurements. Figure 5a,b display clear and stable SCM data and resistance signals with high signal/noise ratio in the different regions. It is worth mentioning that SSRM cannot discriminate the doping type. However, comparison with SCM data makes it possible to identify the different regions. Beneath the AlN layer (Fig. 5a), different colors associated simultaneously to different doping type (n: negative signal or p: positive signal) and doping level (absolute value) can be seen because SCM data are proportional to the product of dC/dV-phase and dC/dV-amplitude. These different colors in SCM data correspond to the *p*-type Si region, space charge region and *n*-type Si region. In the case of resistance measurements with SSRM (Fig. 5b), a dark “burgundy red” area associated to Al and/or Ga diffusions into *n*-type Si substrate is seen below the AlN/Si interface. The border of this dark “burgundy red” color inside the *n*-type Si substrate corresponds to the interface between a *p*-type Si region (or p-type channel) and space charge region. A careful examination of the images demonstrates that the *p*-type region beneath the AlN layer is better resolved with SSRM than with SCM. This is probably due to the difference in spatial resolution between these two techniques. Both techniques highlight the presence of a *p*-type channel below the interface, thereby confirming the C-V and Hall effect measurements as discussed previously.

**Figure 6:**
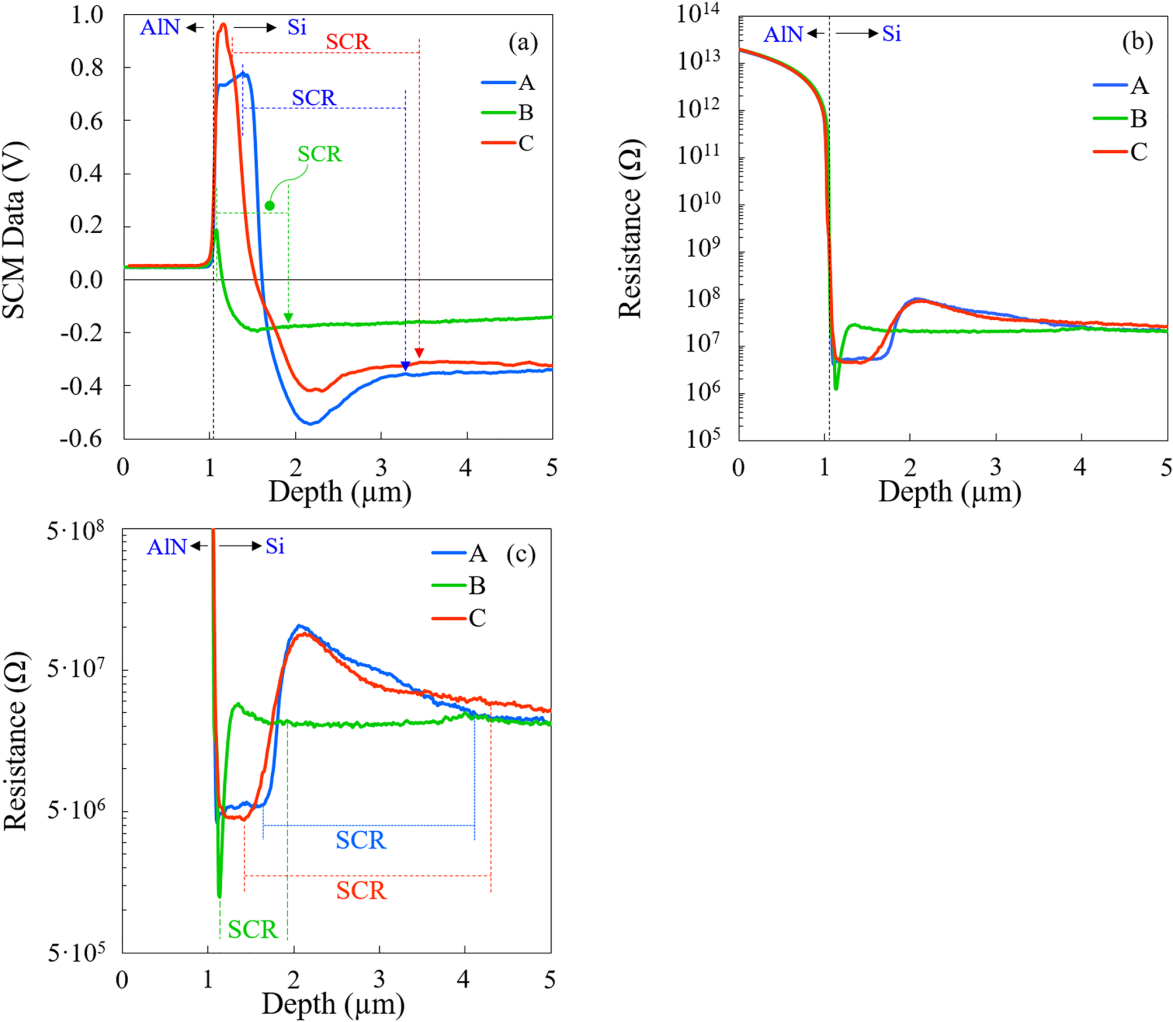
The different plots are obtained by averaging the scan lines that formed the 2-D images for sample A, B and C. (**a**) SCM data show both the type (*n*-type < 0 deg. while *p*-type > 0 deg.) and level of dopants (absolute value) at each point. (**b**) Resistance profile and (**c**) zoom around the space charge region (SCR).

As the measurements are performed on the beveled surface, the lateral dimension has to be converted in order to get back the original vertical dimension. All data in Fig. 6 are plotted as a function of the equivalent depth after conversion and each curve corresponds to a mean value of 1280 scan lines. It should be mentioned that after conversion the equivalent thickness for the AlN side is not equal to ~ 200 nm but corresponds to ~ 1 µm. The latter includes approximately 0.8 µm originating from the surface of the AlN layer (figures S1 and S3). Indeed, to better visualize the beveled surface of the AlN buffer and highlight its electrical activity, the scanned area is slightly extended towards the original surface. The dC/dV-phase and dC/dV-amplitude signals as well as the standard deviations of SCM data are shown in supplementary figures S5 and S6.

SCM data (or dC/dV signal) are displayed in Fig. 6a. The AlN layer exhibits low SCM data of ca. 50 mV corresponding to the SCM electronic noise. This shows that the AlN film is not electrically active, as expected. Note that there is no discontinuity in the SCM data signal because the AlN original surface displays very low RMS (Table 1) necessary for AFM electrical studies as in the case of the beveled samples. Below the interface, positive and negative SCM data are associated with *p*- and *n*-impurity types, respectively. On the one hand, sample B exhibits a *p*-type channel across a shallow depth when compared with samples A and C. On the other hand, samples A and C display a well-defined *p*-type channel with a nearly constant SCM signal while sample B shows a fine peak with significantly lower signal near the interface, even after angle beveling. This is probably due to the fact that the width of the *p*-type channel for sample B is roughly of the same order of magnitude as the depletion region thickness under the SCM-PIT-V2 tip. This observation demonstrates that an AlN buffer deposition at low temperature is effective for inhibiting acceptor dopant diffusion into the *n*-type Si substrate. The position where dC/dV = 0 V is ascribed to the electrical junction which is an interface inside the space charge region where n-doped and p-doped sides have balanced influences on the tip. On a beveled sample, the electrical junction is disturbed due to the redistribution of the free carriers known as carrier spilling relative to the cross-section under the influence of the applied tip bias. In principle, the electrical junction position cannot be used for an accurate determination of the diffusion length (*d*) of the acceptor dopants inside the host *n*-type semiconductor, since its position does not coincide with that of metallurgical junction.

For further information, the *pn-*junction is analyzed using SSRM, which is a resistive measurement method. A positive bias of ca. 2.5 V is applied to the sample while the metallic tip is at virtual ground. Figure 6b,c present the resistance profile for samples A, B and C. As expected, the AlN layer shows resistance as high as 1 × 10^13^ Ω highlighting its insulating properties. Beneath the interface, the resistance drops down to 4.5 × 10^6^ and 1 × 10^6^ Ω in a short depth, then reaches a maximum value of 2.8 × 10^7^ and 1 × 10^8^ Ω before stabilizing at 2 × 10^7^ Ω far from the interface for samples A and B, respectively. Note that sample C displays a similar trend as sample A. The lower resistance confirms that a conductive channel is present beneath the AlN layer. This channel is *p*-type as indicated by SCM. Sample B exhibits a fine SSRM dip very close to the interface and the width of the *p*-type channel is very small, i.e. is roughly of the same order of magnitude as the DDESP-V2 tip radius of curvature, which makes its estimation very difficult. This observation is in agreement with that of SCM and highlights that low temperature deposition of the AlN buffer is beneficial for reducing the acceptor dopant diffusion into the Si substrate. On the other hand, samples A and C show a wider and well-defined conductive *p*-type channel, that is several times larger than the tip radius of curvature, making it possible to extract their physical characteristics without difficulty. As the resistances of the three samples are of the same order of magnitude, the width of the conductive *p*-type channel may be the main factor that controls the physical properties of the GaN-on-Si devices at microwave frequencies. Otherwise, the observed resistance maximum is ascribed to the metallurgical junction due to the space charge region with only few carriers. As for the electrical junction in the case of SCM, the metallurgical junction peak is influenced by the carrier spilling effect, leading to a flattened rather than sharp peak. On the other hand, *p*- and *n*-type regions outside the space charge region show high conductivities, as they are a reservoir of majority carriers. Stangoni^22^ has demonstrated that the SSRM resistance profile shows fine peak in the close vicinity of the metallurgical junction if the positive bias applied to the tip is lower than the cut-in voltage of the *pn*-junction. However, there is always a peak when the tip bias is negative and whatever its absolute value, which is ascribed to the asymmetry in the doping concentration. Otherwise, the resistance of *n*-type Si region is ca. 1000 larger than that of *p*-type Si region outside the *pn*-junction. This is because the *p*-type channel underneath the interface is more heavily doped than the *n*-type Si substrate, which is expected.

Let us now consider the space charge region method. Table 2 summarizes important parameters of the *pn*-junction extracted from SCM data and SSRM profiles. It is worth mentioning that the borders of the space charge region are not known with high precision, especially for the *n*-type side. The border in the *n*-type *(or p*-) side is roughly defined as the position where the SSRM (or SCM) signal becomes stabilized or constant. The position of the electrical junction is located at ca. 0.56 ± 0.08 µm, 0.12 ± 0.08 µm and 0.49 ± 0.08 µm while that of the metallurgical junction is around 1.02 ± 0.08 µm, 0.33 ± 0.08 µm and 1.12 ± 0.08 µm from the AlN/Si interface inside the *n*-type Si substrate for samples A, B, and C, respectively. If these values of metallurgical junction are reported on the SIMS profile, it can be deduced that the involved concentrations of [Ga] and [Al] are of the order of few 10^15^ cm^-3^. Otherwise, the depletion width W of the *pn*-junction at equilibrium with SSRM (SCM) is ca. 2.49 (1.85), 0.76 (0.87) and 2.86 (2.16) for samples A, B and C, respectively. This slight discrepancy may be attributed to the difference in spatial resolution between SCM and SSRM as shown previously.

**Table 2:** Parameters of the *pn*-junction extracted from SCM, SSRM and SIMS profiles.

Techniques	A		B		C	
	SCM	SSRM	SCM	SSRM	SCM	SSRM
W (µm)	1.85	2.49	0.87	0.76	2.16	2.86
W_p_ (µm)	0.62	0.39	0.29	0.23	0.88	0.72
W_n_ (µm)	1.23	2.10	0.58	0.53	1.28	2.14
*d*_EJ_ (µm)	0.56	–	0.12	–	0.49	-
*d*_MJ_ (µm)	–	1.02	–	0.33	-	1.12
*d*_Ga_ (µm) (SIMS)	0.85		0.7		1.0	
*d*_Al_ (µm) (SIMS)	1.6		0.7		1.5	

W = W_p_ + W_n_ is the space charge region width where W_p_ and W_n_ are the width of the *p*-side and *n*-side depletion regions. The constant *d*_EJ_ and *d*_MJ_ are the depth of electrical junction and metallurgical junction location with respect to AlN/Si interface. The symbols *d*_Ga_ and *d*_Al_ are the diffusion depth of Ga and Al atoms obtained by SIMS.

In summary, SCM and SSRM make it possible to establish a 2D profile of the conductive *p*-type channel beneath the interface for the three samples. This *p*-type channel is more pronounced for high temperature deposition and is uniform in depth over a long range very close to the interface. At this stage, these results confirm the C-V measurements i.e. the impurity types of the conductive channel beneath the AlN/Si interface. However, the carrier concentration in the *p*-type channel cannot be estimated with SCM and SSRM due to the lack of a calibration sample. However, the acceptor dopant concentration in the *p*-type side may be estimated using the equation of charge neutrality N_a_ × W_p_ = N_d_ × W_n_^23^, where W_p_ and W_n_ are the width of the *p*- and *n*-type side depletion regions. Calculations are not easy due to the fact that the acceptor and donor concentrations are not constant within the space charge region. Nevertheless, volume hole concentrations will be estimated in the next section based on the sheet resistance and the *p*-type channel widths.

## Discussion

**Figure 7:**
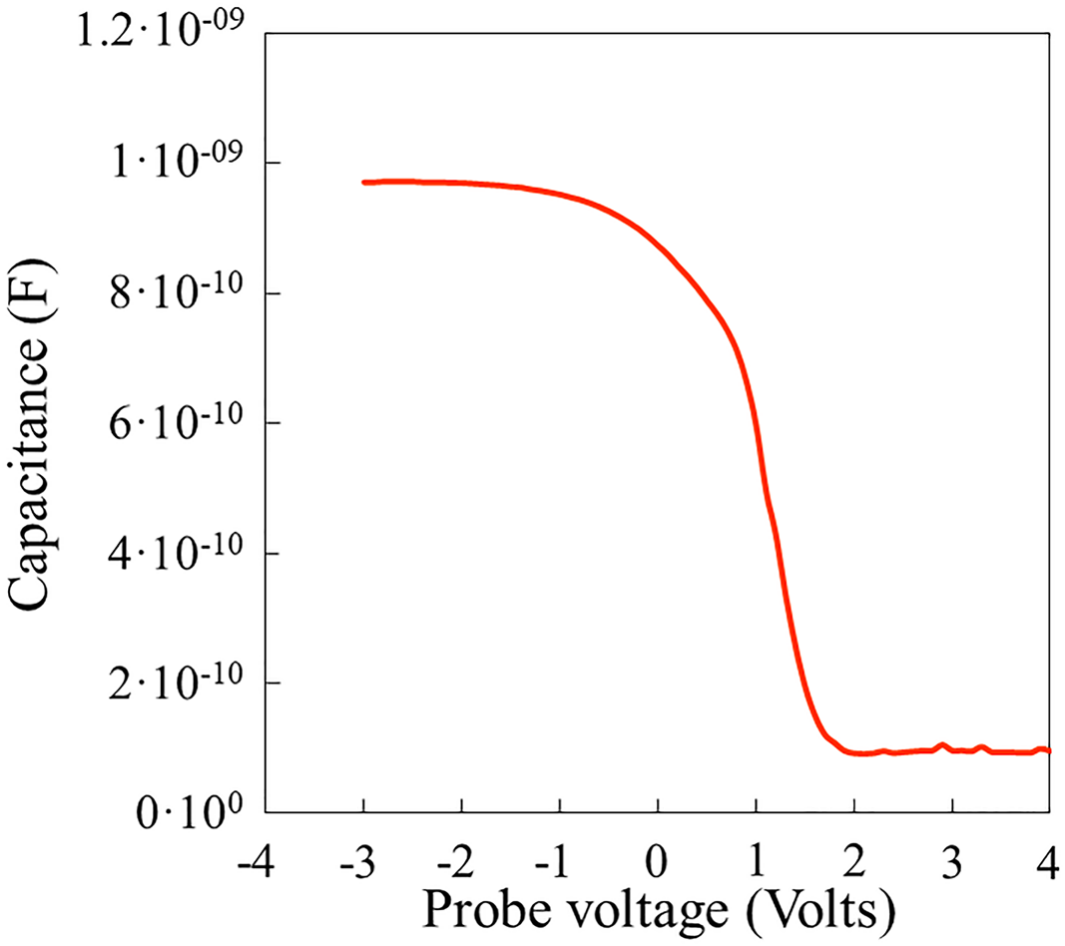
C–V measurements at 10 kHz of the 20 nm grown AlN on Si.

The goal of this work is to understand overall RF propagation losses that affect performance of GaN-on-Si microwave power devices. These losses are actively influenced by any carriers present in the substrate that behave as a resistive power load coupled via the capacitance of the insulating buffer layer. The higher the resistivity, the lower the power dissipated should be. Hence, to limit propagation losses, the holes in the *p*-type channel beneath the AlN/Si interface as well as the free electrons in the *n*-type Si substrate should be as small as possible. Table 3 summarizes some important physical characteristics for samples A, B and C. For each sample, the measured propagation losses vary by less than 20% between 10 and 40 GHz, which means that the absorbing load is efficiently coupled and located quite close to the transmission line. Furthermore, as expected, losses increase when the sheet resistances decrease. At 40 GHz, losses are 5.7, 0.7 and 2 dB/mm for samples A, B and C respectively, while the sheet resistances are 980, 9560 and 3190 ohms/sq respectively. From Hall effect measurements performed on thin AlN sample grown at 900 °C by molecular beam epitaxy^24^, the residual sheet resistance of the Si substrate is of few tens of kOhms, *n*-type. Thus, the sheet resistance of samples A, B and C accounts for the presence of carriers induced by the MOVPE process. C-V measurements indicate that these carriers are holes located in the vicinity of the AlN/Si interface with a volume concentration of few 10^18^ cm^-3^. SIMS measurements revealed that such holes may originate from the diffusion into the substrate of Al and/or Ga behaving as acceptors. As recently reported by Chang et al.^5^ with a more accurate analysis tool (TOFSIMS), the diffusion of such species can be spatially limited to few tens of nanometers while producing noticeable changes in the conductivity into the substrate. In our study, the great majority of Al and Ga atoms are found by the SIMS analysis within the upper 200 nm region of the substrate where concentrations of these elements drop down to lower but detectable amounts beyond. The SCM and SSRM analyses provide the necessary cross section electrical picture of the studied system. In particular, the resistance mapping confirms the presence of a *p*-doped channel close the AlN/Si interface for the three studied samples. This indicates that the lower capacitance measured previously on sample B is not due to a deeper depletion. Furthermore, the width of the *p*-doped channel can be estimated. SSRM profiles indicate that the conduction of holes mainly occurs within 529 nm, 95 nm and 407 nm thick channels for samples A, B and C respectively. Combined with the sheet carrier concentrations previously calculated, these channel widths lead to the volume hole concentrations reported in Table 3. Interestingly, these values in the range of several 10^17^ cm^-3^ vary in a same way as the acceptor concentration determined by C-V and in the range of several 10^18^ cm^-3^. A first explanation for this discrepancy is that the activation energy of acceptors is about 70 meV and not negligible compared to the thermal energy kT = 25 meV and then only a part of acceptors is ionized to generate free holes in the flat band part of the channel. On the other hand, C-V probes the upper part of the channel where the dopant concentrations are high but drop down rapidly; this makes accurate calculations difficult. The SCM and SSRM also show the presence of a *pn*-junction formed between the *p*-doped region and the low *n-*doped Si substrate. The presence of a space charge region depleted of carriers may be seen as interesting for achieving high resistivity. It can be seen that the space charge region is wider in samples A and C compared with sample B. However, in the present case, it is clear that an extended space charge region is concomitant with a higher amount of diffused species, with a resulting increase of conduction close to the interface. Another interesting point in the discussion is the difference between samples A and C. Recall that samples A, B and C were successively grown after a unique reactor preparation consisting of heating the susceptor of the system and injecting gas like hydrogen and ammonia. According to another recent work^19^, Gallium is stored in the showerhead of the CCS reactor. Prior to the reactor preparation that must be considered inefficient, samples with GaN layers had been grown, explaining the observed memory effect. Sample A and sample C were grown with identical conditions but they present noticeably different sheet resistances and RF losses connected with a drop of acceptor and hole concentrations by a factor of about 2. As shown by SCM and SSRM, this mainly affects the vicinity of the AlN/Si interface and the diffusion of species towards deeper regions has much less effect as shown by the similar capacitance and resistance profiles of these samples. From these observations, we conclude that there is a benefit of proceeding to a sacrificial AlN deposition sequence prior to the desired growth on Si. The progressive coating of the showerhead with polycrystalline AlN may reduce the quantity of Gallium that is likely to diffuse towards the substrate. On the other hand, sample B shows the benefit of the combination of this effect with a reduced growth temperature. This is however detrimental in terms of surface roughness but does not clearly affect the crystal quality mainly determined by the nucleation process that was unchanged in the present study. The last point concerns the possibility of achieving the inversion resulting in an electron gas at the AlN/Si interface as mentioned by Yacoub et al.^6^. The C-V measurements performed on the 20 nm nucleation layer previously studied to evidence the *p*-doped region by Hall effect is shown in Fig. 7. The accumulation of holes at the interface is observed for negative bias with a saturation of the capacitance at a level that is about the half of the one expected for 20 nm AlN. On the other hand, the drop of capacitance at positive bias confirms that the depletion of holes is effective in this case but as explained in ref 6 the expected observation of the inversion capacitance is influenced by the fact that the large depletion width makes the capacitance very low and the latter dominates the C-V signal. However, it is worth noticing that compared with the latter study the inversion seems positively shifted by about 2 V, which may be explained by the presence of a larger *p*-type doping level at the interface. As shown in Fig. 6a, the huge drop of *p*-type SCM signal at AlN/Si interface between samples A, C and sample B indicates that the growth temperature and reactor preparation play a major role in the resulting doping. It is expected that further optimizations may also lead to *p*-type doping levels sufficiently low at the interface to permit the depletion and the inversion to occur in the negative bias range.

**Table 3:** Physical characteristics for sample A, B and C: N_a_-N_d_ is the net acceptor concentration, W_h_ is the width of the *p*-type channel and R_sh_ is the sheet resistance.

	Sample A	Sample B	Sample C
N_a_-N_d_ (/cm^3^) from C-V	4 × 10^18^	1.6 × 10^18^	1.9 × 10^18^
W_h_ (µm) from SSRM	0.530	0.095	0.407
Holes (/cm^3^)	4.0–4.7 × 10^17^	2.2–2.7 × 10^17^	1.6–1.9 × 10^17^
R_sh_ (Ohm/sq.)	980	9560	3190
Hole density (/cm^2^) from Hall	2.1–2.5 × 10^13^	2.1–2.6 × 10^12^	6.5–7.8 × 10^12^
Losses at 10 GHz (dB/mm)	5.0	0.6	1.7
Losses at 40 GHz (dB/mm)	5.7	0.7	2.0

## Conclusion

This work demonstrates that a *p*-type conductive channel as well as a *pn*-junction are created beneath the AlN/Si interface after AlN film deposition by MOVPE in a close coupled showerhead system. Chemical (SIMS) and electrical (SCM, SSRM) profiling approaches combined with techniques such as C-V measurements, Eddy current and Hall effect make it possible to fully characterize the AlN/Si interface. On one hand, SIMS investigations demonstrate acceptor dopant in-diffusions (both Al and Ga) due to the process conditions (temperatures/durations/gasses) leading to a metallurgical junction inside the *n*-type Si substrate. The great majority of Ga and Al dopants is mainly located in the first 200 nm beneath the interface. On the other hand, SCM highlights that all samples exhibit *p*-type impurities up to a certain depth beneath the interface, thus forming a conductive channel. This *p*-type channel is more pronounced for high temperature deposition and is quite uniform in depth over a large distance forming a plateau very close to the interface. Furthermore, high signal to noise ratios obtained for the SCM and the SSRM profiles make it possible to identify the space charge region, electrical junction as well as the metallurgical junction beneath the AlN/Si interface. SCM and SSRM highlight that the AlN buffer is not electrically active and has high resistivity. Otherwise, the volume hole concentrations deduced from the sheet carrier densities obtained by Hall effect are of the order of several 10^17^ cm^-3^. These values are one order lower than the few 10^18^ cm^-3^ acceptor concentrations determined by C-V measurements, which is probably due to the fact that acceptors are not totally ionized. It is worth noticing that samples which were grown with the same conditions show discrepancies in the *p*-type channel width, space charge region as well as in the sheet resistances and RF Losses in spite of the reactor preparation. These differences are attributed to the memory effect associated to previous sample growths with GaN. However, the low temperature deposition (i.e. at 1000 °C) of the AlN films allows the *p*-type channel and the *pn*-junction over a shallow depth inside the *n*-type Si substrate to be obtained, minimizing parasitic channel conductivity as well as propagation losses in devices to be fabricated on GaN-on-Si. These promising results constitute a key step in the development of efficient devices at low cost.

## Methods

The samples were grown by Metalorganic Vapor Phase Epitaxy (MOVPE) in a Aixtron Close Coupled Showerhead (CCS) system. The graphite susceptor coated with SiC is heated by three concentric tungsten filaments. The crystalline quality of the layers was assessed by X-ray diffraction on a Panalytical X’PERT Pro MRD system and AFM scans were performed with a Nanoscope IV Dimension 3100 system using tapping mode to avoid any surface degradation. The thickness of AlN layers was determined by grazing incidence X-ray diffraction. SIMS was performed with O_2_^+^ ions and positive ions detection to obtain Al and Ga acceptor dopant chemical profiles underneath the AlN/Si interface. As schematized in Fig. 1a, prior to SIMS measurements, the whole AlN layer was removed using Cl_2_/Ar/CH_4_ reactive ion etching (RIE) in order to suppress the influence of Al from this latter. Indeed, when sputtering with the ion beam from the AlN layer towards the Si substrate, stray signal induced by the “push-in effect” due to crater wall contamination from the top side adds to the signal of the bottom part, thus overestimating the Al concentration underneath the AlN/Si interface.

The electrical parameters are measured on samples without any etching of the surface. Capacitance–Voltage measurements are performed with a MDC Mercury probe (792 µm diameter central dot) connected to an Agilent 4234A LCRmeter. Contactless sheet resistance was measured using a setup based on Eddy current phenomena. RF transmission lines are fabricated using a lift-off process with electron beam evaporation of a 100 nm Ti / 400 nm Au metal stack. Scattering parameters S_ij_ were measured from 0.25 GHz up to 67 GHz using a Vector Network Analyzer (VNA) (Agilent Technologies E8361A) on lines with different lengths to assess the propagation losses.

SCM and SSRM measurements are performed using a Bruker Dimension Icon AFM setup provided with a Nanoscope V controller and equipped with SCM and SSRM application modules. A conductive Pt-Ir coated Sb-doped Si tip (model SCM-PIT-V2) with spring constant of 3 N/m and tip radius of 25 nm is used to carry out the nanoscale capacitance measurements. During the measurements, the MIS structure formed by the conductive tip with AlN/Si is biased with a high frequency (i.e. 90 kHz) voltage of 2500 mV through the chuck. Maximum dC/dV signal is obtained with DC offset equals to 0 V. The applied force to the tip is as low as 72 nN and the scan rate is set to 0.25 Hz. The working frequency of the capacitance sensor is ca. 915 MHz. For nanoscale resistance measurements (SSRM), a conductive diamond coated Sb-doped Si tip with spring constant of 80 N/m and tip radius of 100 nm (model DDESP-V2) is used and a DC bias of 2.5 V is applied to the sample. The force applied to the tip is set to 4 µN and the scan rate is the same as that for SCM. A SSRM module integrating logarithmic amplifier capable of sensing currents ranging from 10 pA to 0.1 mA is used. For a fixed bias, the current flows from the tip to the sample chuck through the semiconductor and the resistance is obtained by Ohm’s law (i.e. R = V_DC_/I). For SCM and SSRM electrical modes, the tip was scanned in contact mode.

Sample surface preparation is extremely important for successful SCM and SSRM measurements. SCM spatial resolution is closely linked to the lateral distribution of the depletion region beneath the tip while that of SSRM depends on the tip radius of curvature. The total AlN layer thickness for sample A, B and C is around 200 nm and the tip radius is equal to 25 nm for SCM and 100 nm for SSRM leading thus to 8 and 2 pixels, respectively. An alternative way to enhance the spatial resolution is to expand the area of interest by angle beveling. The sample preparation is as follows: (i) samples are cleaved along the $[11\overline{2}0]$ crystal direction of GaN. After that, the native oxide film on the back side of the *n*-type Si substrate is removed by RIE with the following parameters at room temperature: working pressure of 3 mTorr, 430 W RF power, 5 sccm He / 3 sccm C_2_H_4_ / 30 sccm CHF_3_ gases and time of 5 s. Immediately after, a back-side contact is deposited by sputtering a thin gold layer of 200 nm. (ii) Samples are mounted on a bevel block using melted wax. They are lapped with bevel angle of ca. 10° (× 5.76 magnification of depth dimension) using diamond discs with grain size of 3 and 1 µm and alumina discs with grain size ranging from 0.3 to 0.05 µm. (iii) Afterward, (a) polished samples are immersed in HF (50%): H_2_O (ratio of 1: 10 in volume) solution for 30 s to remove the native oxide and (b) a good quality oxide is obtained by a dip in H_2_O_2_ (30%) during 20 min at room temperature^25^. Samples are glued on a metal bevel holder with silver paste in such a way that the beveled surface is parallel to probed plane (figure S1). For SSRM measurements where the oxide film is not necessary, the same procedure is used except the last oxidation step iii·(b) and then the measurements under air are performed immediately after iii(a). The measurement time per sample did not exceed 1 h in order to minimize the effect of air oxidation (so called native oxide), which reappears inevitably. At the end, the bevel angle is measured using a confocal microscope (figure S2).

## Supplementary information

Supplementary information.
