# Supplementary material for: Electrical activity at the AlN/Si Interface: identifying the main origin of propagation losses in GaN-on-Si devices at microwave frequencies
Source: Sci Rep. 2020 Aug 25;10:14166. doi: 10.1038/s41598-020-71064-0 (PMC7447785; doi:10.1038/s41598-020-71064-0)
Supplement: Supplementary file 1 — Supplementary information. [file 41598_2020_71064_MOESM1_ESM.pdf]

# Electrical activity at the AlN/Si Interface: identifying main origin of the propagation losses in GaN-on-Si devices at microwave frequencies


Micka Bah[1], Damien Valente[1], Marie Lesecq[2], Nicolas Defrance[2], Maxime Garcia Barros[2], J-C. De Jaeger[2], Eric Frayssinet[3], Rémi Comyn[3], Thi Huong Ngo[3], Daniel Alquier[1], Yvon Cordier[3]

[1] GREMAN UMR-CNRS 7347, Université de Tours, INSA Centre Val de Loire, 16 rue Pierre et Marie Curie, BP 7155, 37071 Tours Cedex 2, France
[2] CNRS-IEMN – Université de Lille, UMR8520, Av. Poincaré, 59650 Villeneuve d'Ascq, France
[3] Université Côte d'Azur, CNRS, CRHEA, rue B. Gregory, 06560 Valbonne, France

**Corresponding authors**: daniel.alquier@univ-tours.fr, yvon.cordier@crhea.cnrs.fr


## Supplementary Information

**Figure S1** displays the sample configuration used during SCM and SSRM measurements. Let define *d* and *D* as metallurgical junction positions in the cross-section and beveled surface, respectively. The equivalent depth in the cross-section after conversion is $d = D \times sin\alpha$, where $\alpha = 10°$ is the bevel angle The experimental error on *D* can be estimated as follow: the lateral scale (here: 40 µm) of the image is divided by 512 pixels composing it in the lapping direction and then by $1/sin\alpha$ = 5.76. Thus, the spatial resolution after angle beveling is of 14 nm, which is approximately half of the SCM tip dimension (25 nm).

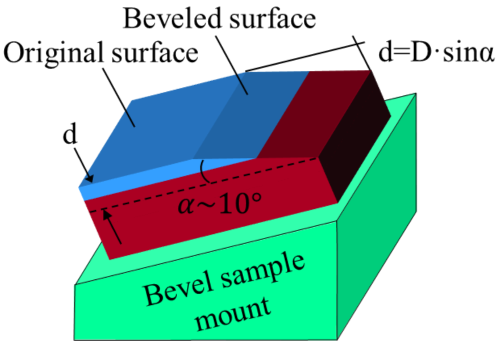


**Figure S1**: sample configuration during SCM and SSRM measurements

**Figure S2** shows the angle beveling for sample A, B and C after cross-section polishing. The angle is checked with confocal microscope equipped with software image treatment. The angle 10° corresponds to a magnification factor of ~ 5.76 in the lapping direction.

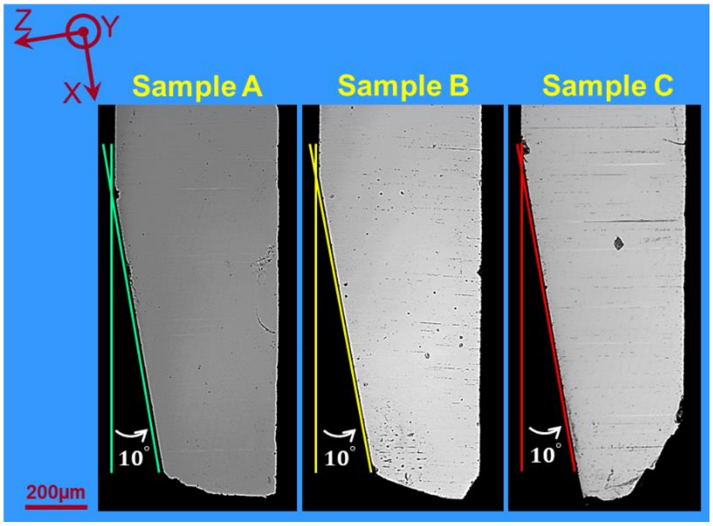


**Figure S2**. Angle beveling for sample A, B and C that was checked with confocal microscope.

**Figure S3** displays topography images such as height and deflection error, which provide information about roughness and interfacial junction between AlN layer and Si substrate for sample A, B and C. Note that, the scan area is extended to the original surface in order to better visualize the beveled area of AlN buffer and to highlight its electrical activity. Therefore, the equivalent depth (~1 µm) in the cross-section after lateral dimension conversion for AlN buffer does not coincide with ~ 200 nm but includes that of the original surface. Otherwise, the sample surface is uniform and displays low roughness (see section 3.2). At the AlN/Si interfacial boundary, a shallow crevice, which appeared as a white line, is evidenced after a careful examination of the deflection error images. The crevice is due to the difference in mechanical properties between AlN layer and Si substrate. At this location, the contact area between the tip and the sample may increase inducing thus a high SCM signal. However, during the measurements, the crevice, which appears during the polishing step, does not affect the measurement, as the SCM signal is very high for the *p*- and *n*-side Si.

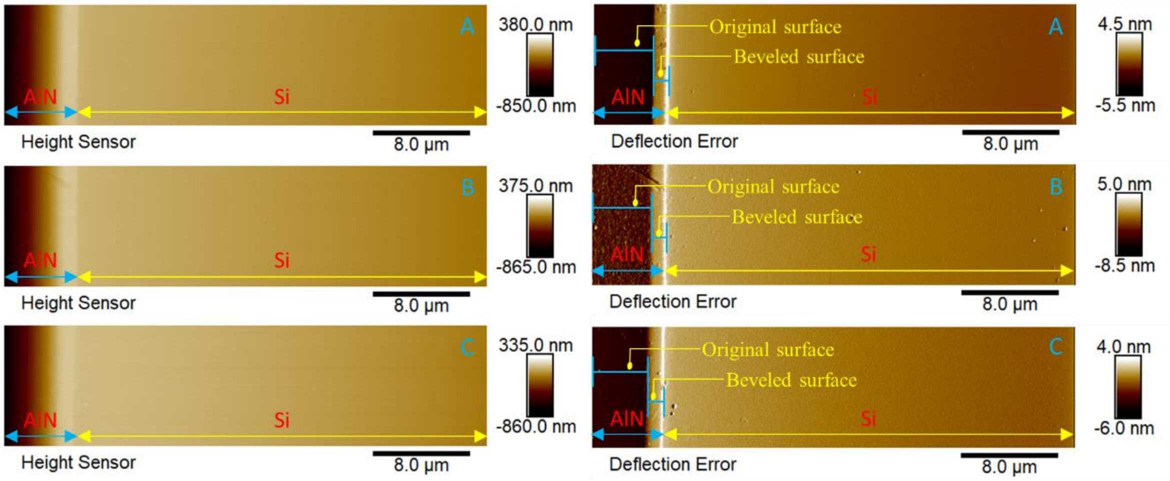


**Figure S3.** Topography and deflection error images for sample A, B and C after angle beveling polishing. AlN/Si interface is visible.

**Figure S4** displays the dC/dV-phase and the dC/dV-amplitude. The dC/dV-phase (positive or negative) is associated to the doping type while the dC/dV-amplitude (always positive) furnishes the doping concentration. Their product leads to SCM Data, which informs simultaneously on doping type and level.

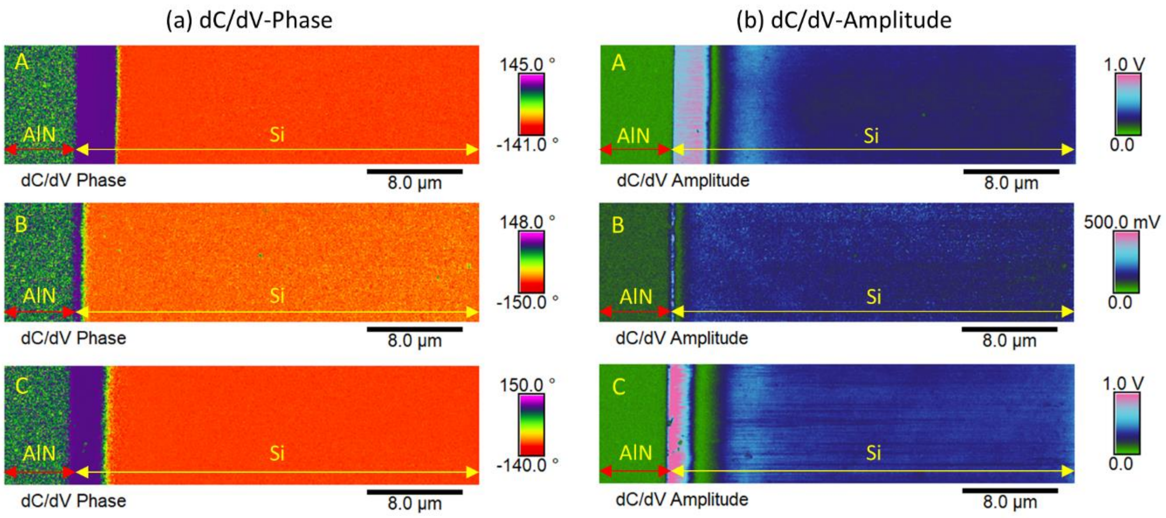


**Figure S4**. (a) dC/dV-phase: green color corresponds to AlN layer while violet and red colors are associated to *p*- and *n*-type Si regions, respectively for sample A, B and C. (b) dC/dV-amplitude provides information about doping concentration for the three different regions cited above.

**Figure S5** displays the profiles of dC/dV-phase and dC/dV-amplitude. Before carrying measurements, a SRAM test sample supplied with the SCM sensor was used to set the lock-in phase. The AlN layer shows positive phase and very low amplitude and their product corresponds to the SCM electronic noise (ca. 50 mV) evidencing that AlN film is not electrically active. Beneath the AlN/Si interface, 90° and -90° phase variations are associated to *p*- and *n*-impurity types, respectively, for sample A, B and C. The dC/dV-amplitude is always positive and gives information about impurity doping concentration. The very sharp peak is ascribed to the EJ or intrinsic interface within the SCR.

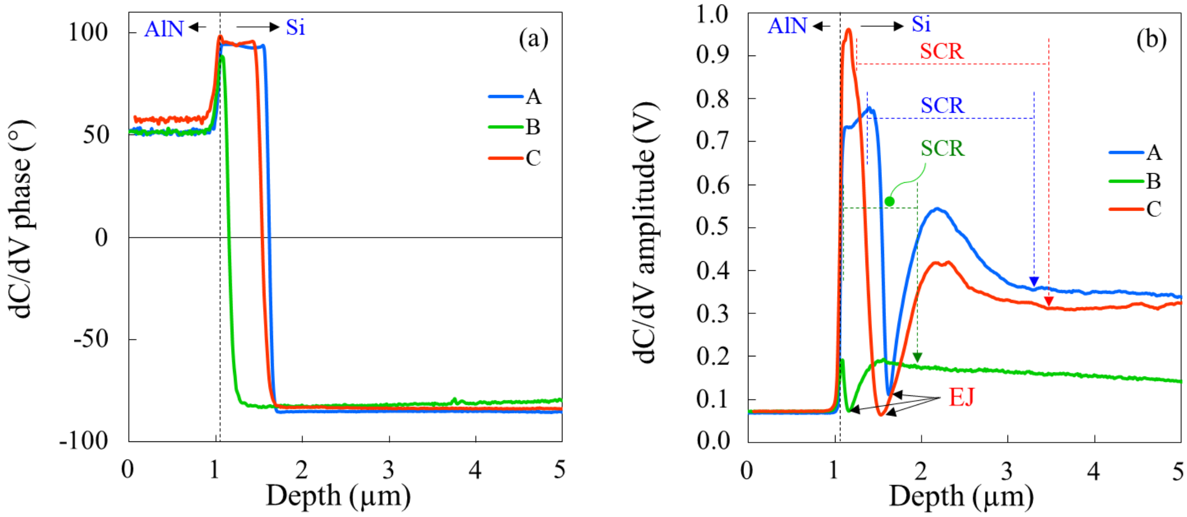

**Figure S5**. The different plots are obtained by averaging the scan lines that formed the 2-D images of the **figure S4**. (a) dC/dV-phase is associated to the doping types (*n*-type < 0 deg. while *p*-type > 0 deg.), (c) dC/dV-amplitude is always positive and informs on the doping concentration.

To confirm the reproducibility of the SCM measurements, a set of 10 measurements with scan size of 40×10 $\mu m^2$ (i.e. 512×128 pixels) was carried out at different locations. A mean value of SCM Data and standard deviation were calculated from 1280 scan lines for each point (i.e. 512) in the lapping direction and the results are plotted in **Figure S6**. Low standard deviations are observed for sample A, B and C evidencing thus the stability of the SCM signal during the measurements.

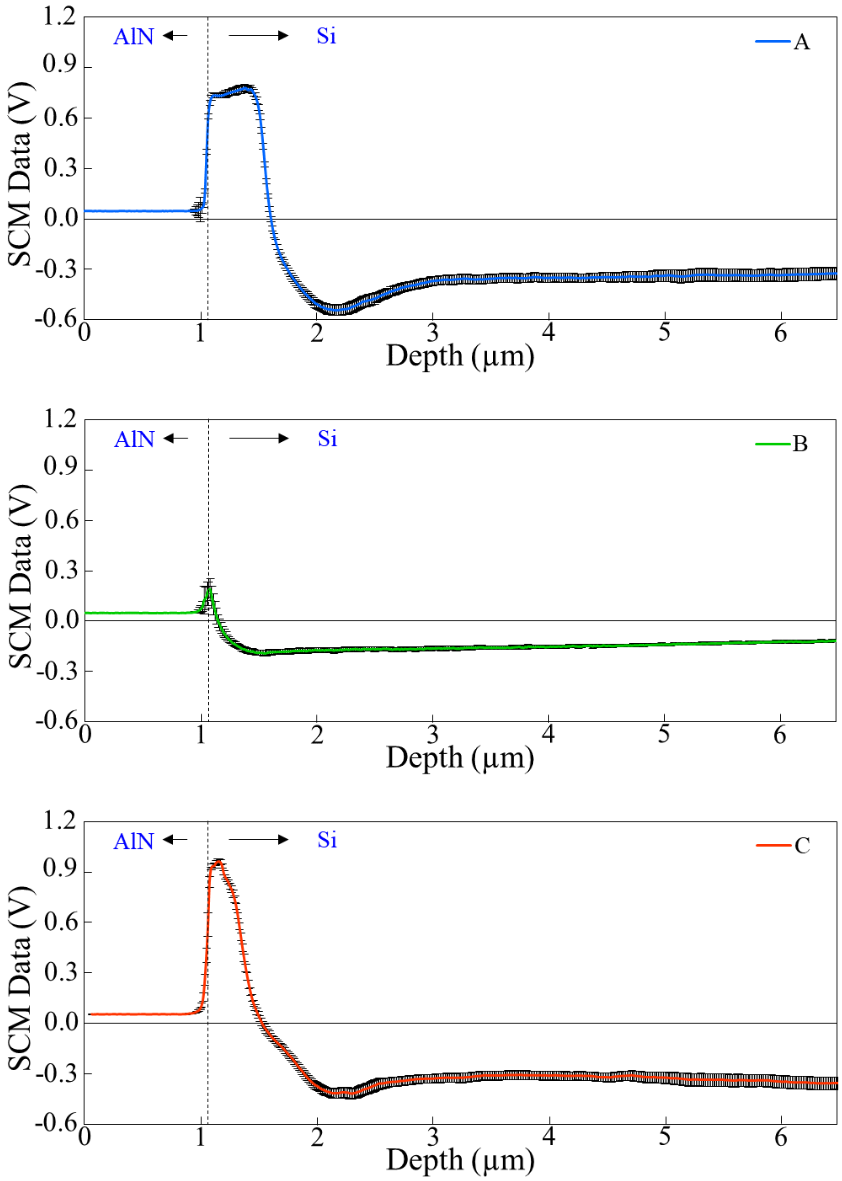


**Figure S6**. Solid lines: mean value of SCM Data from 1280 scan lines and error bars: corresponding SCM Data measurement uncertainties for sample A, B and C.
